# Predictive Performance of Placental Protein 13 for Screening Preeclampsia in the First Trimester: A Systematic Review and Meta-Analysis

**DOI:** 10.3389/fmed.2021.756383

**Published:** 2021-11-19

**Authors:** Yifan Wu, Yang Liu, Yiling Ding

**Affiliations:** ^1^Department of Obstetrics, The Second Xiangya Hospital, Central South University, Changsha, China; ^2^Department of Obstetrics, The Second Affiliated Hospital, School of Medicine, Zhejiang University, Hangzhou, China

**Keywords:** meta-analysis, PP13, preeclampsia, screening biomarker, early-onset preeclampsia

## Abstract

Preeclampsia is a pregnancy-specific syndrome that affects maternal and neonatal mortality. Several serum biomarkers can be used to predict preeclampsia. Among these proteins, placental protein 13 (PP13) has received progressively more interest in recent studies. The decrease in PP13 expression is one of the earliest signs for the development of preeclampsia and has shown its predictive performance for preeclampsia. In this meta-analysis, we collected 17 observational studies with 40,474 pregnant women. The overall sensitivity of PP13 to predict preeclampsia was 0.62 [95% confidence interval (CI) = 0.49–0.74], the specificity was 0.84 (95%CI = 0.81–0.86), and the diagnostic odds ratio was nine (95%CI = 5–15). The area under the curve for summary receiver operating characteristic was 0.84. We then chose the early-onset preeclampsia as a subgroup. The sensitivity of early-onset subgroup was 0.63 (95%CI = 0.58–0.76), the specificity was 0.85 (95%CI = 0.82–0.88), and the diagnostic odds ratio was 10 (95%CI = 6–18). The findings of our meta-analysis indicate that PP13 may be an effective serum biomarker for the predictive screening of preeclampsia. Nonetheless, large prospective cohort studies and randomized controlled trials are expected to uncover its application in clinical practice. The heterogeneity of the original trials may limit the clinical application of PP13.

**Systematic Review Registration:** https://www.crd.york.ac.uk/PROSPERO/display_record.php?RecordID=188948 The meta-analysis was registered in PROSPERO (CRD42020188948).

## Introduction

Preeclampsia is a pregnancy complication that affects 3–5% of pregnant women and causes maternal, fetal, and neonatal mortality (1). Besides the typical high blood pressure, preeclampsia is also characterized by several multi-system damages, including acute kidney injury, liver involvement, and neurological and hematological complications. Furthermore, uteroplacental dysfunctions, including fetal growth restriction, abnormal umbilical artery Doppler waveform analysis, and stillbirth are common symptoms and consequences of preeclampsia (2).

The etiology of preeclampsia remains incompletely elucidated, an increasing number of studies are still investigating the potential predictive screening of preeclampsia, aiming to diagnose it as early as possible (3). Some biomarkers in maternal blood present screening values in early pregnancy, such as maternal placental growth factor, soluble fms-like tyrosine kinase 1, and plasma protein A (4–7). In addition to serum biomarkers, various diagnostic modalities, such as uterine artery Doppler, mean arterial pressure, and maternal history, are meaningful for predictive screening of preeclampsia (8–10). Furthermore, recent studies have reported the predictive value of placental protein 13 (PP13) (8, 11–13).

PP13, also known as Galectin 13, LGALS 13, is one of the 56 known placental proteins. It binds to annexin IIa, β-galactoside, and β/γ actin, suggesting its multiple activities during the entire pregnancy (14). PP13 is important for embryo implantation, maternal-fetal immune tolerance, placental development, and vascular remodeling (15–18). Quantification methods of PP13 include enzyme-linked immunosorbent assay and dissociation-enhanced lanthanide fluorescence immunoassay. In a healthy pregnant woman, serum levels of PP13 increase slowly with gestational age; however, a lower level of PP13 is detected during the first trimester in patients who are later diagnosed with preeclampsia, indicating the possible predictive function of PP13 for screening preeclampsia in asymptomatic women (19). In addition, PP13 is the earliest changed molecule related to preeclampsia, which indicates its specific advantages compared with other known biomarkers (20). In this meta-analysis, we searched for clinical studies on PP13 testing that predicted preeclampsia individually to review the predictive testing of PP13 in this disease.

## Materials and Methods

### Search Strategy and Study Selection

**Figure 1:**
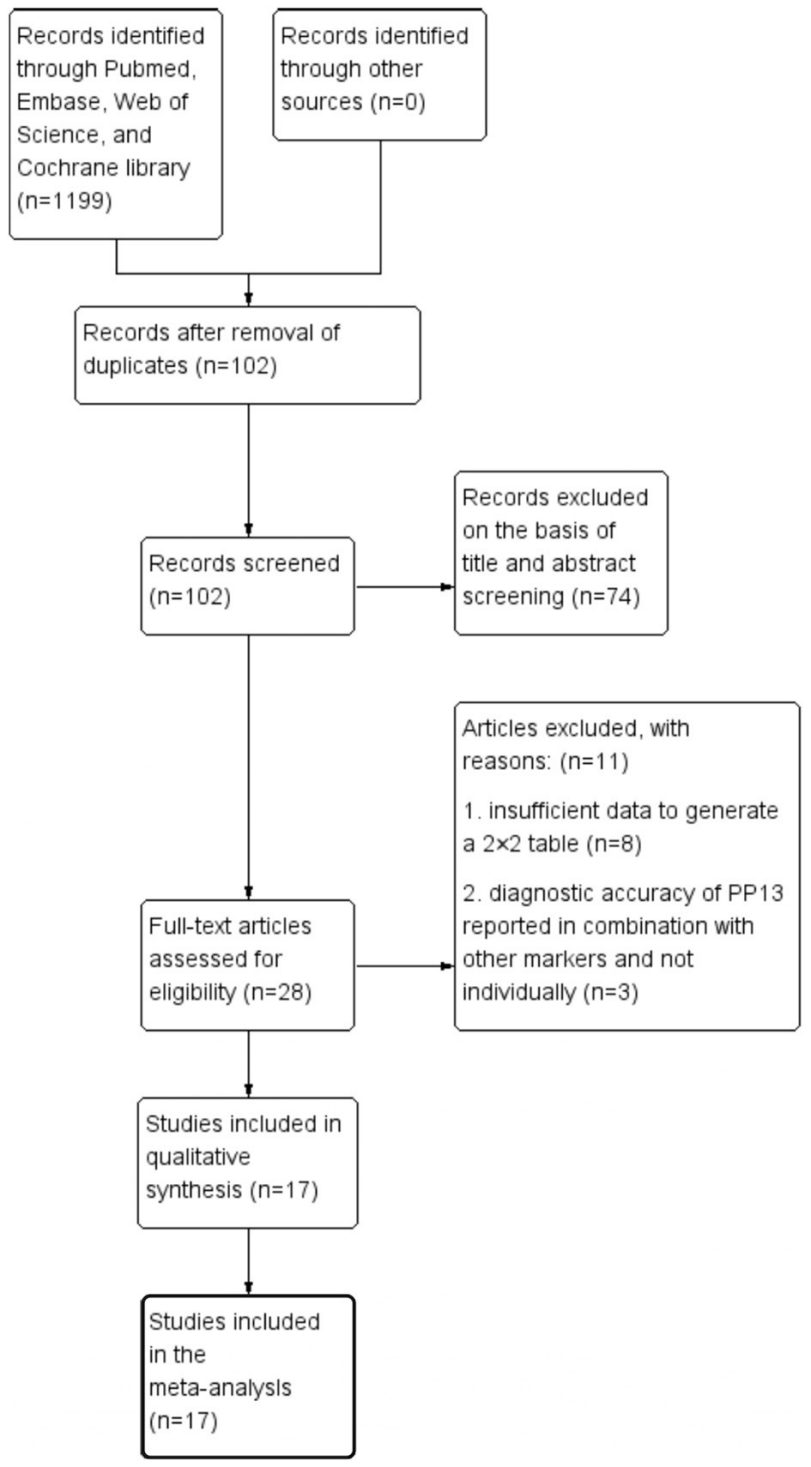
Search strategy and study selection as per Preferred Reporting Items for Systematic Reviews and Meta-Analyses for Protocols guidelines.

This meta-analysis was performed according to the Preferred Reporting Items for Systematic Reviews and Meta-analyses for Protocols 2015. After searching PubMed, Embase, Web of Science, and Cochrane library databases, the reference lists of all primary articles and reviews were examined to identify papers cited by electronic searches. The strategy contained MeSH terms, Emtree terms, and some key words of preeclampsia and PP13. The entire process of study selection consisted of four stages: identification, screening, eligibility, and inclusion (Figure 1). Identification of studies meant searching the articles in the database according to the search strategy. This process was completed by two reviewers (YW and YL) individually, and the third reviewer decided the final list if there were any disagreements with the identification of studies. The reviewers then screened the cited studies individually using the title and abstract. In the final stage, the full texts of papers were collected to finish the quality assessment and finally obtain a 2 × 2 table for analysis. The meta-analysis was registered in PROSPERO (CRD42020188948).

### Inclusion Criteria for the Studies

Inclusion criteria were as follows:

1. Research on pregnant women studying PP13 in blood samples, including serum and plasma for the prediction of preeclampsia.
2. Studies including cross-sectional, case-control, and cohort studies.
3. At the time of screening, pregnant women had no signs or symptoms of preeclampsia.
4. Studies only including singleton pregnancies.
5. Studies published in English.
6. Research published until April 31, 2020.

### Exclusion Criteria for the Studies

Exclusion criteria were as follows:

1. Data not available to construct a 2 × 2 table to calculate the diagnostic values of the test.
2. Data reported in combination with other markers as predictive models instead of PP13 alone.
3. Data presented on the outcomes, including other diseases and the part of preeclampsia, which could not be analyzed independently.

### Data Extraction and Study Quality Assessment

**Figure 2:**
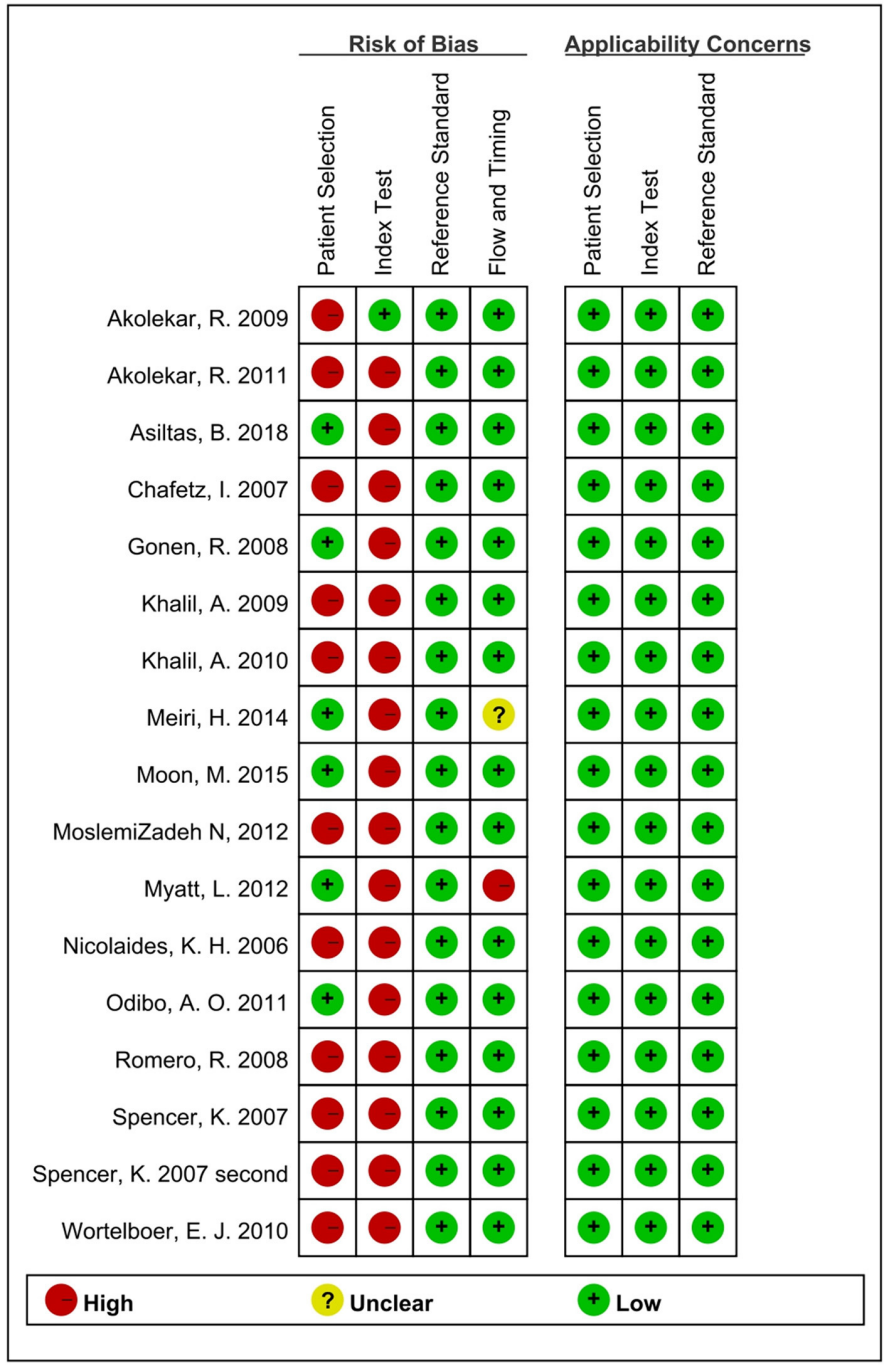
Quality analysis of included studies.

The data about the research were extracted individually by two reviewers (YW and YL) according to a standard protocol. Any disagreements in data extraction were resolved by a third reviewer. The extracted data included the published details (the year of publication, first author, country of trials, and published journal), characteristics of the study (type of study design, population of research, number of preeclampsia and no preeclampsia groups, gestational screening, and mainly outcomes of different subgroups), and the data needed to construct a 2 × 2 table. The methodological quality of all studies included in the meta-analysis was checked by one of the reviewers (YW) according to QUADAS-2 (Figure 2). The tool was recommended for use in systematic reviews and meta-analyses to assess the risk of bias and applicability of primary diagnostic accuracy studies. The key points of bias included patient selection, index test, reference standard, flow, and timing.

### Data Synthesis and Analysis

The data are shown in the 2 × 2 diagnostic table, including the numbers of true positives, false positives, false negatives, and true negatives. According to the table, the sensitivity, specificity, and likelihood ratios were calculated with 95% confidence interval (CI). To show the results more clearly and directly, the diagnostic odds ratio was chosen as a combined indicator to explain and compare the effects of prediction. As a traditional tool, the receiver operator characteristic curve (ROC) and area under the curve were also chosen to show the performance of the screening test. The heterogeneity of the meta-analysis was checked using Cochran *Q* and *I*^2^ statistics. If there was significant heterogeneity, a random-effects model was applied, and the identification was *p* < 0.10 or *I*^2^ >50%. For hierarchical models, the hierarchical summary ROC (HSROC) was used to show equal summary estimates after considering variability from the heterogeneity and random sampling error. The Der Simonian-Laird method was used for the estimation of random effects. HSROC was reported as the x-axis (sensitivity) and y-axis (specificity). Furthermore, the area under the HSROC curve was calculated to indicate the strength of the relationship between the predictive test and disease. To analyze the possible sources of heterogeneity, a meta-regression was performed. The parameters of the regression included the preeclampsia group size, total group size, and country. Some cited articles not only provided the main outcome (preeclampsia) data, but also reported the results of subgroups, such as early-onset preeclampsia (EO-PE). Furthermore, various clinical studies have indicated an association between PP13 and preeclampsia, especially EO-PE (21–23). Therefore, we identified EO-PE as a subgroup to show the possible difference in strength of the predictive effects of general preeclampsia and EO-PE. Publication bias was checked using Deek's funnel plot asymmetry test.

All data were statistically analyzed using Review Manager 5.4 (RevMan, Version 5.4 for Windows, Nordic Cochrane Center, Copenhagen, 2020) and StataSE 15.0 (StataCorp, College Station TX).

## Results

### Identification and Quality Assessment

Figure 1 shows the process of literature identification and selection. After searching the databases, we obtained 1,199 articles related to PP13 and preeclampsia. After removing duplicates, there were 102 articles. Of these papers, 74 were basic research articles and reviews. Of all the clinical trial studies, 11 articles did not meet the selection criteria. After selection, identification, and quality assessment, 17 articles were included in the meta-analysis (19, 20, 22, 24–37). The quality assessments of these studies are summarized in Figure 2. In the final studies (*n* = 17), there were 11 articles organized as the case-control study design, and all papers were good at consecutive and random patient enrollment, index test results, reference standard, and flow and timing.

### Study Characteristics

Table 1 summarizes the study characteristics of the 17 studies. The countries of these studies included the USA (*n* = 4), UK (*n* = 8), Turkey (*n* = 1), Netherlands (*n* = 1), Iran (*n* = 1), and Israel (*n* = 2). Regarding the populations, three articles showed that they focused on a priori high-risk women, and one study chose the low-risk population. The definition of a priori high-risk pregnant women includes chronic hypertension, pre-gestational diabetes mellitus, chronic renal disease, systemic lupus erythematosus, antiphospholipid syndrome, obesity (body mass index ≥30 kg/m^2^), or a history of preeclampsia in the previous pregnancy. The cited paper did not provide criteria for a low-risk population. Gestational age at the time of screening was between 8 and 14 weeks. Furthermore, three studies reported the results of screening in the second trimester and/or third trimester. Given that the number of studies (*n* = 3) was small to provide enough statistical power, we did not collect these data as subgroups in this study. However, there were some studies that calculated more than one related outcome (*n* = 7), such as total preeclampsia (the type of preeclampsia not distinguished), EO-PE, and late-onset preeclampsia (LO-PE). In this situation, we collected only the total preeclampsia data to avoid duplication. Nine articles reported the results for EO-PE; hence, we analyzed this part individually as a subgroup. Two studies also showed the outcome of LO-PE, but we did not analyze this outcome in the analysis because the number of studies was small to provide enough power (*n* = 2). A few articles (*n* = 7) chose preeclampsia as the only outcome. Only one study identified the subgroup as severe and mild preeclampsia, so we did not analyze this subgroup situation. Regarding the cutoff value of PP13, most studies (*n* = 5) showed the cutoff as the relationship with multiples of the median, one article chose 71.8 pg/mL as cutoff, another article chose 88.5 pg/mL, and other articles (*n* = 10) did not provide specific cutoff values. All studies did not predetermine the cutoff; rather, it was decided according to the ROC with maximal accuracy. Additional details are provided in Table 2.

**Table 1:** Study characteristics.

**No**.	**Published year**	**Authors**	**Country**	**Study design**	**Population**	**Cutoff value**	**Outcomes**	**Preeclampsia (*n*)**	**No preeclampsia (*n*)**
1	2018	Asiltas, B.	Turkey	Cohort studies	General population	≤71.8	PE	38	122
2	2015	Moon, M.	USA	Cohort studies	General population	Didn't show	PE	50	468
3	2014	Meiri, H.	Israel	Cohort studies	General population	0.4 MOM	PE	63	757
4	2012	Moslemi Zadeh, N.	Iran	Case–control study	General population	≤88.5	PE	100	100
5	2012	Myatt, L.	USA	Cohort studies	Low risk	Didn't show	PE	174	509
6	2011	Odibo, A. O.	USA	Cohort studies	General population	Didn't show	PE and EO	42	410
7	2011	Akolekar, R.	UK	Case–control study	General population	Didn't show	EO, IO, and LO	752	32,850
8	2010	Wortelboer, E. J.	the Netherlands	Case–control study	General population	Didn't show	PE	88	480
9	2010	Khalil, A.	UK	Case–control study	Priori high risk	Didn't show	PE and EO	42	210
10	2009	Khalil, A.	UK	Case–control study	Priori high risk	0.66 MOM	EO, IO, and LO	42	210
11	2009	Akolekar, R.	UK	Case–control study	Priori high risk	Didn't show	EO	48	416
12	2008	Romero, R.	USA	Case–control study	General population	0.39 MOM	EO, severe, and mild PE	50	250
13	2008	Gonen, R.	Israel	Cohort studies	General population	0.4 MOM	PE	20	1,178
14	2007	Spencer, K.	UK	Case–control study	General population	Didn't show	PE, EO, and LO	44	446
15	2007	Spencer, K.	UK	Case–control study	General population	Didn't show	PE and EO	24	144
16	2007	Chafetz, I.	UK	Case–control study	General population	0.38 MOM	PE	47	290
17	2006	Nicolaides, K. H.	UK	Case–control study	General population	Didn't show	EO	10	423

**Table 2:** Overview of study characteristics.

**Study characteristics**	**Number of studies**
**Country**	
USA	4
UK	8
Turkey	1
The Netherlands	1
Iran	1
Israel	2
**Patient population**	
A priori high risk	3
Low risk	1
General population	13
**Study design**	
Case control study	11
Others	6
**Gestational age of screening**	
<14 weeks	17
14–20 weeks	1
≥20 weeks	3
**Type of outcome**	
Identified as EO and/or LO	9
Identified as mild and/or severe PE	1
Didn't identified sub-groups	7
**Type of cutoff value**	
Accurate value	2
Relationship with MOM	5
Didn't report cutoff	10

### Data Analysis

**Figure 3:**
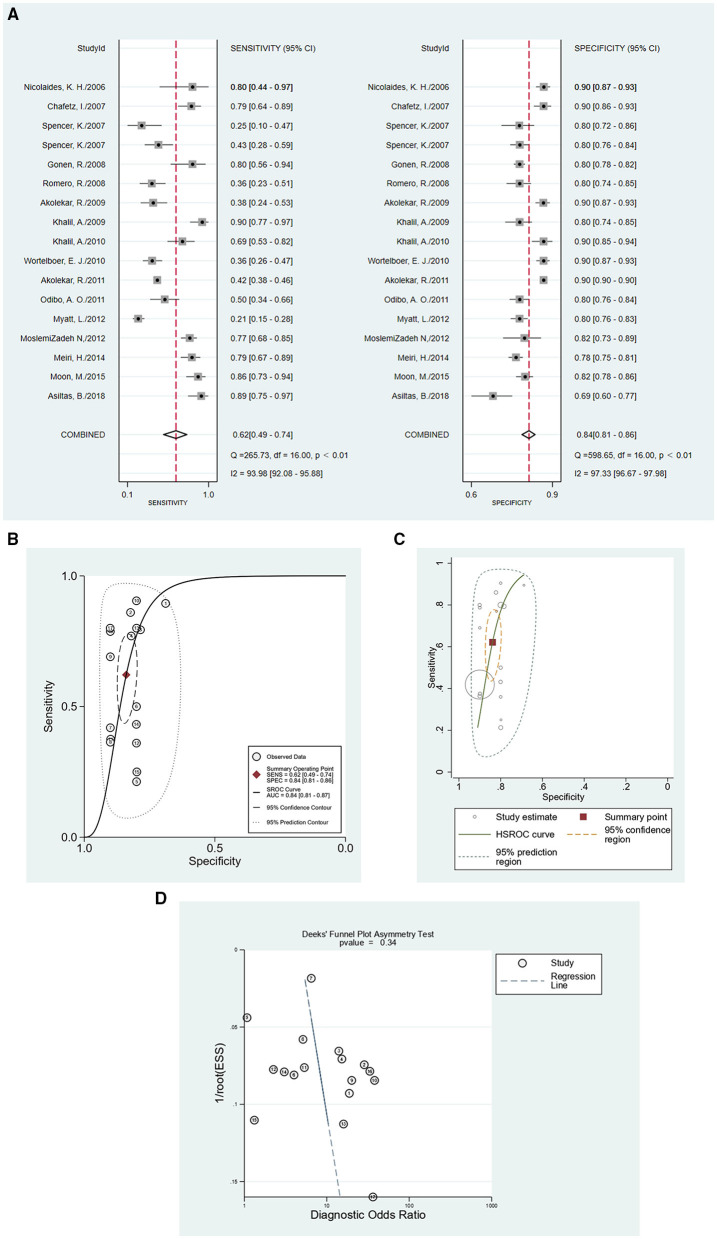
The accuracy of PP13 as demonstrated by **(A)** forest plot, **(B)** SROC curve for all included studies, **(C)** HSROC curve for all included studies, and **(D)** publication bias test for all included studies.

**Figure 4:**
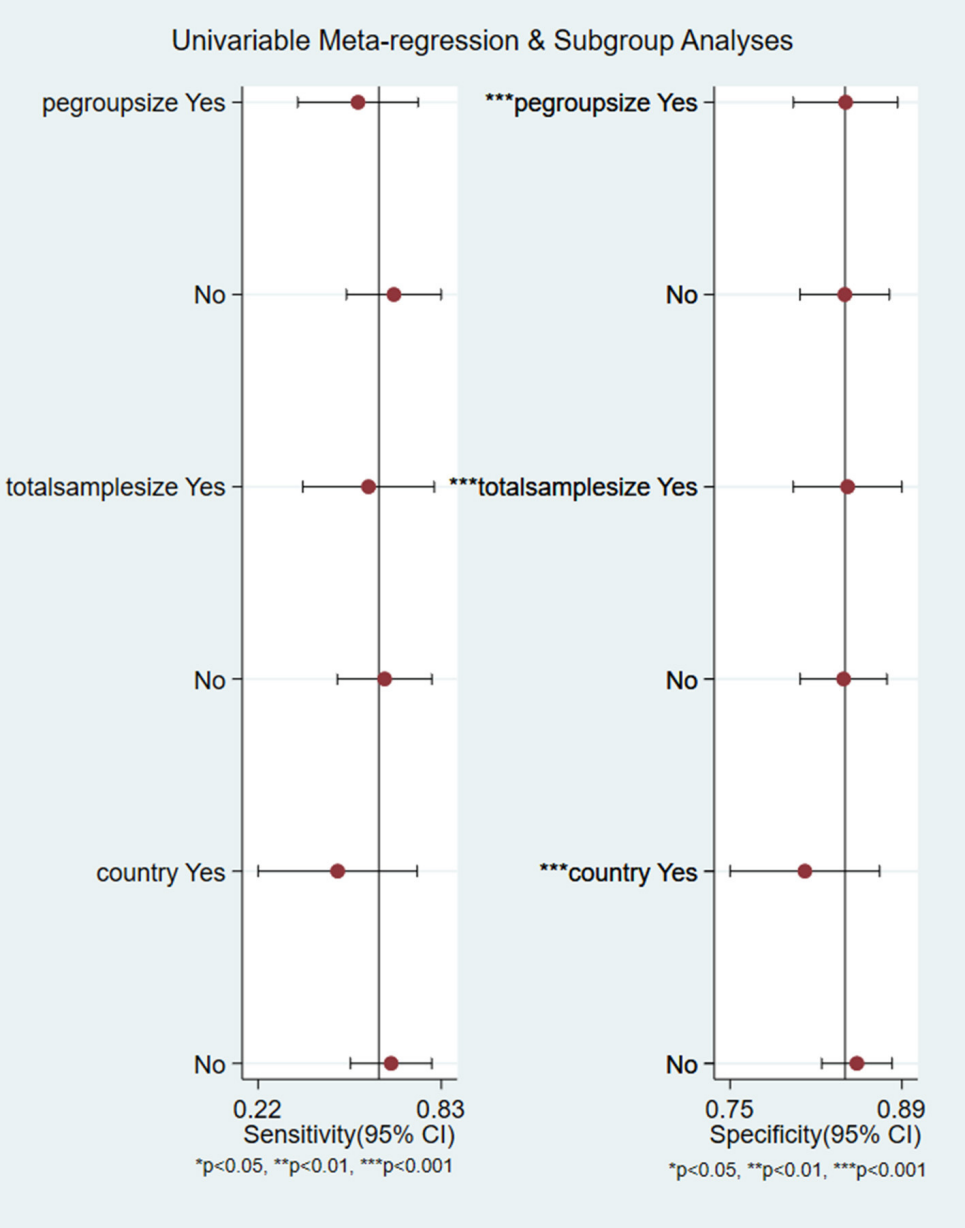
Meta-regression analysis for all included studies. *p < 0.05, **p < 0.01.

In this meta-analysis, 17 studies were included with a total of 40,474 pregnant women, of whom 1,634 were preeclampsia patients and 39,263 were controls. The sensitivity and specificity of all cited research was 0.62 (95%CI = 0.49–0.74), and 0.84 (95%CI = 0.81–0.86), respectively. The positive likelihood ratio was 3.9 (95%CI = 3.0–4.9), and the negative likelihood ratio was 0.45 (95%CI = 0.32–0.63). The diagnostic odds ratio was nine (95%CI = 5–15). Figures 3A,B show the forest plot and summary ROC for all studies (*n* = 17). The random-effects model was chosen because of the high heterogeneity induced by the different clinical and methodological studies. The area under the curve for summary ROC was 0.84 (95%CI = 0.81–0.86). Figure 3C shows the HSROC, suggesting the ROC type of trade-off between sensitivity and specificity, indicating a threshold effect. It was difficult to analyze the bias of different cutoff values chosen by different studies, which in most articles did not report the accuracy value. The heterogeneity of the collected trials may be a limitation of this application. There were no randomized controlled trials in the meta-analysis. The publication bias test is shown in Figure 3D (*p* = 0.34), which shows that there was no bias caused by the publication. As mentioned above, we used meta-regression analysis to identify the source of the high heterogeneity. The results of the analysis are shown in Figure 4. According to the results, the preeclampsia group size, total sample size, and country of study localization can affect the outcome of the specificity.

**Figure 5:**
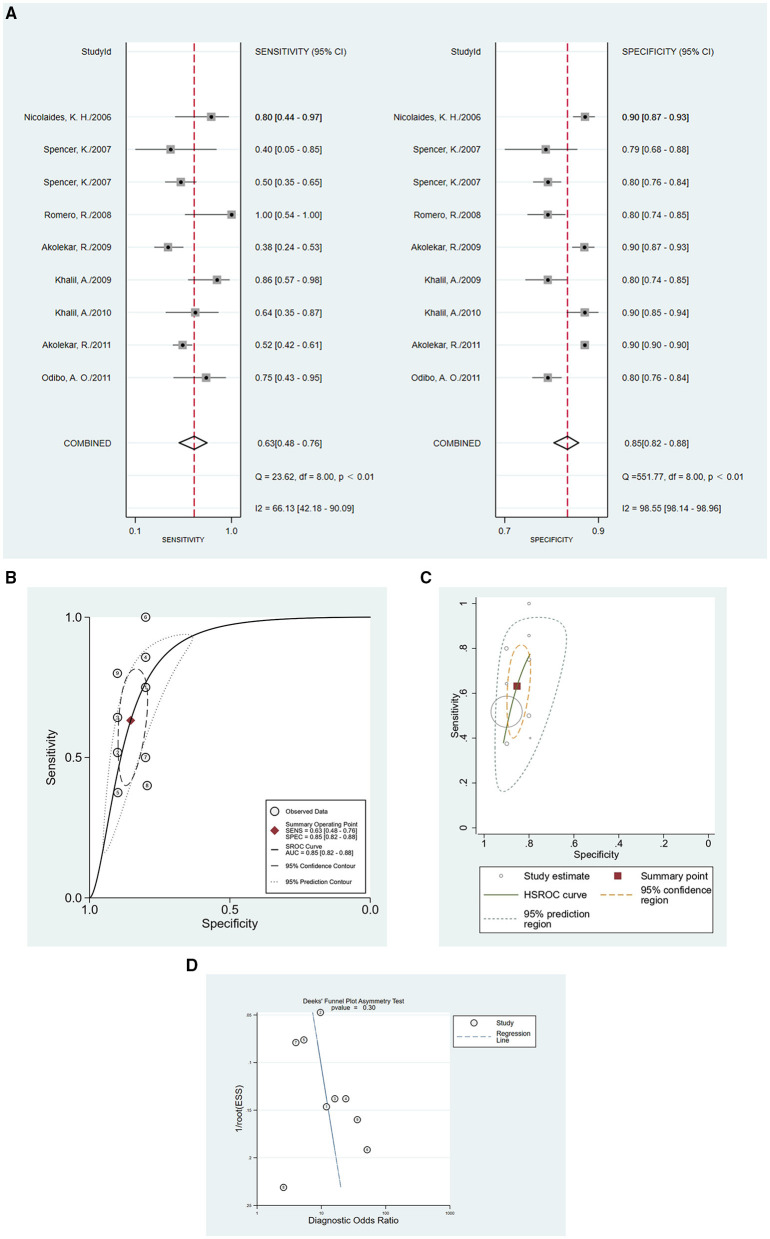
The accuracy of PP13 as demonstrated for EO-PE by **(A)** forest plot, **(B)** SROC curve for EO-PE group, **(C)** HSROC curve for EO-PE group, and **(D)** publication bias test for EO-PE group.

EO-PE has recently received increasing interest (38–41). In our meta-analysis, there were a few articles that reported data on EO-PE as the individual outcome (*n* = 9), so we analyzed those parts as subgroup data. In the EO-PE group, the sensitivity of studies was 0.63 (95%CI = 0.58–0.76), the specificity was 0.85 (95%CI = 0.82–0.88), positive likelihood ratio was 4.3 (95%CI = 3.3–5.6), negative likelihood ratio was 0.43 (95%CI = 0.30–0.62), and the diagnostic odds ratio was 10 (95%CI = 6–18). Figures 5A,B show the forest plot and summary ROC for the EO-PE group. The area under the curve for summary ROC was 0.85 (95%CI = 0.82–0.88). The HSROC is presented in Figure 5C. The publication bias test is shown in Figure 5D. There were not enough clinical trials of LO-PE; hence, we could not obtain credible results regarding the difference in predictive performance between EO-PE and LO-PE, or between EO-PE and total preeclampsia. Various studies have reported that some molecules, including serum levels of soluble LIGHT, serum autotaxin, serum high temperature requirement A1, FVIIa-antithrombin, and soluble trigger receptor expressed on myeloid cells-1, have shown different concentrations in EO-PE and LO-PE (42–46). Further studies are necessary to confirm the differences in PP13 levels between the two subtypes of preeclampsia.

## Discussion

In this meta-analysis, we included 17 studies to examine the predictive performance of PP13 in the diagnosis of preeclampsia. The results indicate that PP13 may be a powerful predictive biomarker for preeclampsia screening.

### PP13 and Preeclampsia

There are an increasing number of studies investigating the etiology of preeclampsia, although the underlying mechanisms remain unclear (47–51). Nevertheless, numerous biomarkers have been demonstrated to be potential predictive molecules, including the anti-angiogenic factor soluble fms-like tyrosine kinase-1, the angiogenic factor PGF, plasma protein A, and PP13. Several meta-analyses have examined their predictive effects for preeclampsia separately (4, 7, 52), while no meta-analysis has investigated the predictive role of PP13.

The messenger ribonucleic acid and protein expression levels of PP13 were significantly reduced in case of preeclampsia (15), and this change in PP13 during the progression of preeclampsia can be tested via maternal peripheral blood. According to studies on PP13, PP13 is secreted from an early stage of pregnancy and can be detected in the serum as early as the 5th week of gestation (53), which helps us to test it at the beginning of antenatal care.

PP13 plays a vital role in pregnancy and plays a role in the development of preeclampsia. In the immune regulatory network, PP13 showed an essential role in regulating the activity of neutrophils, which reduced the apoptosis rate of neutrophils and increased the expression of programmed death-ligand 1, hepatocyte growth factor, tumor necrosis factor-α, reactive oxygen species, and matrix metalloproteinase 9 toward a placental-growth-permissive phenotype (54). In preeclampsia, the lower concentration of PP13 cannot express the immunoregulatory phenotype, which leads to the failure of trophoblast growth and invasion. It is notable that PP13 does not affect the functionality of neutrophils, such as neutrophil extracellular trap release, degranulation, phagocytosis, and bacterial reactive oxygen species response. In addition, PP13 functions in immunoregulatory processes, including induction of apoptosis of activated T cells and killing of decidual macrophages (55).

Successful spiral artery remodeling is necessary for normal placental development. Studies that examined the relationship between PP13 and maternal vessels also described that PP13 plays a key role in the expansion and dilation of the uterine vasculature in animal models (56, 57). The possible mechanism underlying this relationship can be explained by the endothelial signaling pathway (58). Given the vital function of PP13 in pregnancy, it is reasonable to examine its predictive performance in preeclampsia.

### Type of Preeclampsia

The classification of preeclampsia as early-onset and late-onset, rather than mild or severe is of clinical significance (2). The difference between EO-PE and LO-PE not only lies in the timing, but also exists in the pathophysiology and clinical implications. Studies on preeclampsia have suggested that EO-PE is a disease of placental origin (41). Nonetheless, LO-PE may mark abnormal interactions between the placenta and genetics of maternal cardiovascular and metabolic diseases. In the two-stage pathogenesis of preeclampsia, the first stage is defective trophoblast invasion, which leads to stressed placenta; in the later stage, the placental syncytiotrophoblast affects the whole-body system and results in the failure of clotting regulation, fluid transfer, and blood pressure regulation. Therefore, based on the pathogenesis of EO-PE and the function of PP13 in the placenta, PP13 may confer a stronger predictive performance in EO-PE. Therefore, we chose EO-PE as the subgroup for analysis. According to the clinical data collected, there were not enough data about LO-PE, so we could not compare the predictive power of PP13 between EO-PE and LO-PE.

### Distant Cutoff Values and Other Confounders

The cutoff values of PP13 varied across studies, but most studies did not report accurate cutoff values. The lack of a standardized cutoff value not only limited the clinical applicability of PP13 in screening tests, but also increased the heterogeneity of the meta-analysis. The difference in testing methods is one factor that can affect the applicability of screening tests. Of the studies included in this analysis, most studies used enzyme-linked immunosorbent assay and dissociation-enhanced lanthanide fluorescence immunoassay to test PP13 in maternal peripheral blood samples. Consequently, it is necessary to identify unanimous testing methods and the cutoff value of PP13 to predict preeclampsia in the first trimester. In addition, the basic characteristics of patients, such as age, ethnicity, parity, body mass index, and smoking status, should be considered. In combination with the basic characteristics, the biomarker PP13 may have a more significant predictive performance.

### Advantages and Limitations of the Study

To the best of our knowledge, this meta-analysis is the first to summarize the existing articles on the predictive performance of PP13 for preeclampsia. In our analysis, 17 studies with total 40,474 pregnant women were included by the inclusion criteria, exclusion criteria, and quality assessment by QUADAS-2 tool. With regard to heterogeneity, we used meta-regression and extensive subgroup analyses to minimize the effect of confounders. In addition to the index that we analyzed, 11 studies chose the case-control study design, which caused selection bias. Furthermore, most articles did not report the accurate standard of PP13; hence, there was a threshold effect in the analysis. The different populations and outcome classifications may also affect the efficiency of the predictive performance of PP13 for screening preeclampsia in asymptomatic women.

### Implications for Current Clinical Practice and Future Research

To date, the newly onset symptoms of hypertension and proteinuria remain the most useful screening criteria for preeclampsia, as these are among the first symptoms presented in preeclampsia patients, and these symptoms are easy to detect in clinical practice (39). Our analysis indicates that PP13 may act as an effective predictive biomarker for screening of preeclampsia in the first trimester. When compared with other popular predictive molecules of preeclampsia, PP13 is almost the earliest changed biomarker during the development of preeclampsia. According some meta-analyses about individual serum biomarkers screening of preeclampsia, the sensitivity and specificity of plasma protein A is 0.16 (95%CI = 0.04–0.35) and 0.93 (95%CI = 0.76–0.99), respectively (59). For placental growth factor, the sensitivity and specificity of predictive test is 0.78 (95%CI = 0.67–0.86) and 0.88 (95%CI = 0.75–0.95), respectively (4). There is no meta-analysis regarding soluble fms-like tyrosine kinase individually. In our study, the overall sensitivity of PP13 to predict preeclampsia was 0.62 (95%CI = 0.49–0.74), the specificity was 0.84 (95%CI = 0.81–0.86). It may be a powerful screening test if clear cutoff values can be identified in clinical practice. In addition to predicting preeclampsia solely by PP13, it is meaningful to examine the possibility of PP13 combined with other biomarkers and imagological diagnosis. According the recent article regarding PP13 and preeclampsia, the sensitivity has increased from 51.7 (PP13 individually) and 10.3 (uterine artery pulsatility index individually) to 58.6 (combination of PP13 and uterine artery Doppler pulsatility index) (11). In the future, it will be important to explore more models of the combination of PP13 and other biomarkers and diagnostic modalities.

In addition, large-scale prospective cohort studies and randomized controlled trials are needed to report more details regarding the predictive capability of PP13 in different subgroup populations and distant subtypes of preeclampsia.

Although accurate treatments for preeclampsia are often used after the diagnostic results, there is still an increasing interest in identifying or diagnosing this disease at an early stage for identification of those women who may develop preeclampsia and other severe complications in the late pregnancy stage. The reliable results of the predictive test may suggest some additional pregnancy care, which includes more frequent maternity inspection and other supplementary tests, to diagnose and relieve this disease as early as possible. Further, specific therapies should be chosen as soon as the diagnosis of preeclampsia is established, although delivery is usually the only useful treatment. Nonetheless, there are still some precautions, including low doses of aspirin and calcium, along with other replenishing therapies. Diet and lifestyle interventions have also been investigated in previous studies (39). For patients with impaired PP13 molecule or function, replenishing PP13 may be useful for the treatment of preeclampsia (60). A trustworthy predictive screening test could help identify the timing and populations who need supplementary care and preventive measures.

In this analysis, it was shown that PP13, which is expressed at a lower level in the early stage of preeclampsia, has good overall test accuracy for predicting preeclampsia in asymptomatic pregnant women in the first trimester. The significant test for predictive performance in asymptomatic pregnant women indicates the risk for those patients who may develop preeclampsia. Further randomized controlled trials and prospective cohort studies are needed to determine the cutoff value and its application in clinical practice. Furthermore, there is a potential difference in predictive power between EO-PE and LO-PE.

## Data Availability Statement

The original contributions presented in the study are included in the article/Supplementary Material, further inquiries can be directed to the corresponding author.

## Author Contributions

YW was responsible for the initial plan, study design, data collection, data extraction, data interpretation, and statistical analysis. YL was responsible for data collection, data extraction, and statistical analysis. YD was responsible for the study design, data extraction, and statistical analyses. All authors contributed to the article and approved the submitted version.

## Conflict of Interest

The authors declare that the research was conducted in the absence of any commercial or financial relationships that could be construed as a potential conflict of interest.

## Publisher's Note

All claims expressed in this article are solely those of the authors and do not necessarily represent those of their affiliated organizations, or those of the publisher, the editors and the reviewers. Any product that may be evaluated in this article, or claim that may be made by its manufacturer, is not guaranteed or endorsed by the publisher.

## Abbreviations

PP13: placental protein 13
CI: confidence interval
ROC: receiver operator characteristic curve
HSROC: hierarchical summary receiver operator characteristic curve
EO-PE: early-onset preeclampsia
LO-PE: late-onset preeclampsia.
