# Supplementary material for: Predictive Performance of Placental Protein 13 for Screening Preeclampsia in the First Trimester: A Systematic Review and Meta-Analysis
Source: Front Med (Lausanne). 2021 Nov 19;8:756383. doi: 10.3389/fmed.2021.756383 (PMC8640131; doi:10.3389/fmed.2021.756383)
Supplement: Supplementary file 1 [file Table_1.DOCX]

Search Strategies (pubmed)

1 Pre-Eclampsia/

2 pre-eclamp*.tw,kf.

3 pre-clamp*.tw,kf.

4 pre?clamp*.tw,kf.

5 (pregnancy adj3 tox?emia*).tw,kf.

6 1 or 2 or 3 or 4 or 5

7 PP13.tw

8 Placental protein 13.tw

9. Galectin 13.tw

10.LGALS13.tw

11.7 or 8 or 9 or 10

12.6 and 11
